# Comparison of clinical efficacy and safety between interventional embolization and craniotomy clipping for anterior circulation aneurysms

**DOI:** 10.12669/pjms.39.5.7092

**Published:** 2023

**Authors:** Bo Lei, Guoliang You, Xiaoqiang Wan, Honggang Wu, Niandong Zheng

**Affiliations:** 1 Bo Lei, Department of Cerebrovascular Disease, People’s Hospital of Leshan, Leshan 614000, Sichuan, P.R. China; 2 Guoliang You, Department of Cerebrovascular Disease, People’s Hospital of Leshan, Leshan 614000, Sichuan, P.R. China; 3 Xiaoqiang Wan, Department of Cerebrovascular Disease, People’s Hospital of Leshan, Leshan 614000, Sichuan, P.R. China; 4 Honggang Wu, Department of Cerebrovascular Disease, People’s Hospital of Leshan, Leshan 614000, Sichuan, P.R. China; 5 Niandong Zheng, Department of Cerebrovascular Disease, People’s Hospital of Leshan, Leshan 614000, Sichuan, P.R. China

**Keywords:** Interventional embolization, Craniotomy clipping, Anterior circulation aneurysms, Efficacy, Security

## Abstract

**Objective:**

To investigate the clinical efficacy and safety of interventional embolization in the treatment of anterior circulation aneurysms.

**Methods:**

Eighty patients with anterior circulation aneurysms admitted to People’s Hospital of Leshan from June 2019 to December 2021 were retrospectively analyzed. According to the different surgical methods, they were divided into two groups: the observation group and the control group. Patients in the observation group were given interventional embolization, while those in the control group were given craniotomy clipping. The surgical efficacy, postoperative neurological function and quality of life, surgical prognosis and surgical complications of the two groups were compared.

**Results:**

The intraoperative blood loss and hospitalization time in the observation group were lower than those in the control group (*p<*0.05). The scores of the Hunt-Hess and modified Rankin scale in the observation group were significantly lower than those in the control group three months after surgery (*p<*0.05). The good prognosis rate of the observation group was higher than that of the control group (*p<*0.05). Moreover, the complication rate of the observation group was 12.50%, which was significantly lower than 32.50% in the control group (*p*<0.05).

**Conclusion:**

Interventional embolization shows the advantages of minimally invasive procedures such as shorter operative times and shorter hospital stays. It has better clinical safety because it can significantly improve the neurological function and quality of life of patients, improve the prognosis of patients, and reduce the incidence of complications.

## INTRODUCTION

Anterior circulation aneurysms are a common cerebral vascular disease in neurosurgery, which tends to make inroads in middle-aged and elderly people. It mostly occurs in abnormal bulging of the intracranial artery wall and is the primary cause of subarachnoid hemorrhage clinically.^1^,^2^ The etiology of the anterior circulation aneurysm has a close bearing on arteriosclerosis, hypertension and vasculitis.^3^-^5^ Currently, anterior circulation aneurysms are generally treated by early surgery in principle^6^, and craniotomy clipping has long been the preferred surgical method.^7^ Despite the classic operation and definite curative effect, this operation is characterized by large surgical trauma, many postoperative complications, and an unsatisfactory prognosis. With the rapid development of medicine and the appearance of all kinds of medical auxiliary materials, interventional embolization has been applied to the treatment of anterior circulation aneurysms. At present, certain differences still exist about how to choose the above two methods for the treatment of anterior circulation aneurysms in clinical practice. Based on this, in this study, eighty patients with anterior circulation aneurysms were selected as subjects to comprehensively compare the clinical effects of interventional embolization and craniotomy clipping in the treatment of anterior circulation aneurysms.

## METHODS

Eighty patients with anterior circulation aneurysms admitted to People’s Hospital of Leshan from June 2019 to December 2021 were retrospectively analyzed. According to different surgical methods, they were divided into two groups: the observation group (interventional embolization) and the control group (craniotomy clipping), with 40 cases in each group. There was no difference in general data between the two groups (*p>*0.05).

### Ethical Approval:

This study has been approved by the medical ethics committee of Ethical Approval: People’s Hospital of Leshan (No.: [2022]124; date: November 16, 2022), and written informed consent was obtained from all participants.

### Inclusive criteria:

- Patients diagnosed as anterior circulation aneurysms by DSA or CTA.
- Patients who have surgical evidence and have received surgical treatment.
- Patients who provided informed consent to this study and signed the consent form.
- Patients with complete clinical data and active cooperation.

### Exclusion criteria:

- Patients with cerebral infarction and brain trauma.
- Patients with abnormal coagulation function.
- Patients with severe systemic diseases, severe liver and kidney dysfunction, cardiopulmonary dysfunction, and difficulty in tolerating surgery.
- Patients with disturbance of consciousness and mental illness.

### Surgical methods:

Both groups of patients received basic treatment such as preventing vasospasm and relieving pain after admission, and their vital signs were closely monitored. Among them, the patients in the control group were treated with craniotomy clipping. After general anesthesia was successful, the patient’s position was adjusted, the specific position of the aneurysm was located according to the preoperative angiographic results, the surgical area was disinfected, the tissues were separated layer by layer after incision, the bone flap was removed, the cerebral dura mater was cut open, the aneurysm was separated along the subarachnoid space, and the aneurysm was temporarily clipped with a vascular clip. After separation, an appropriate aneurysm clip was selected and clipped, and after hemostasis, the aneurysm was sutured layer by layer. The patients in the interventional group were treated with endovascular interventional embolization. The patient was under general anesthesia, heparinized during surgery, and blood pressure was controlled to maintain systolic blood pressure at 100-110 mmHg. Cerebral angiography was performed first to clarify the location, size and shape of anterior circulation aneurysms. Subsequently, the right femoral artery was punctured, the arterial sheath was indwelled, a super-sliding guide wire and a guide catheter were inserted, the stent catheter was delivered to the distal end of the aneurysm artery, and the microcatheter for embolization was successively reached into the aneurysm sac. According to the size of the aneurysm, the size and length of the coil were selected to effectively fill the aneurysm.

### Observation indicators:

The perioperative-related indicators of the two groups were observed, including operation time, intraoperative blood loss, intraoperative aneurysm rupture and hospital stay. Raymond-Roy grading was used to evaluate the curative effect of the operation, which was divided into four grades: Grade-I: complete embolism, no contrast agent filling in the tumor body and neoplasia neck; Grade-II: visible residues in the neoplasia neck, contrast agent filling showed “dog ear sign”; Grade-III: visible residue in the most of the neoplasia neck, and most of the contrast agent was filled; Grade-IV: visible residue in the tumor body, and the tumor cavity was filled with contrast agent. The National Institute of Health Stroke Scale (NIHSS) and modified Rankin scale were used to evaluate the neurological function and quality of life of patients before and three months after surgery. The higher the NIHSS score, the more serious the neurological deficit of the patient is. The modified Rankin scale scores 0-6, the higher the score, the greater the impact on quality of life. After six months of treatment, Glasgow Outcome Scale (GOS) was used to evaluate the prognosis of the two groups, which were divided into five grades. Among them, 1-3 points indicate a poor prognosis and 4-5 points indicate a good prognosis. The good prognosis rate of the two groups was counted. Moreover, the complications of the two groups were observed, including cerebral vasospasm, rebleeding, cerebral infarction, neurological dysfunction and intracranial infection. The maximum follow-up time for patients in both groups was six months. And case data collection ceased in June 2022. The follow-up work of all patients was completed by the same group of surgeons.

### Statistical Analysis:

All data in this study were statistically analyzed by SPSS 20.0 software, and measurement data were expressed as (*X̅*±*S*). T test was used for preoperative and postoperative comparison. Enumeration data were expressed as n (%), and the c² test was used for the comparison of rates. P<0.05 indicates a statistically significant difference.

## RESULTS

The intraoperative blood loss and hospitalization time in the observation group were significantly lower than those in the control group, with statistically significant differences (*p<*0.05). The proportion of postoperative Raymond-Roy Grade I and II in the observation group was 97.50% (39/40) and 2.50% (1/40), respectively, while that of postoperative Raymond-Roy Grade I and II in the control group was 92.50% (37/40) and 7.50% (3/40), respectively. The proportion in the observation group was slightly higher than that in the control group, but there was no statistically significant difference between the two groups (c²=1.053, *P*=0.305) Table-II

**Table-I:** Comparison of perioperative indicators between the two groups.

Group	Operation time (min,χ̅±S)	Intraoperative blood loss (ml,χ̅±S)	Intraoperative rupture [n,(%)]	Hospitalization time (d,χ̅±S)
Control group (n=40)	141.38±9.67	97.50±19.32	4(10.00)	16.23±1.78
Observation group (n=40)	144.88±6.35	142.25±20.06	6(15.00)	21.55±2.24
*t/χ²* value	1.913	10.163	0.457	11.778
*P* value	0.060	0.000	0.499	0.000

**Table-II:** Comparison of postoperative Raymond-Roy grading between the two groups [n (%)].

Group	Grade I	Grade II
Control group (n=40)	39 (97.50)	1 (2.50)
Observation group (n=40)	37 (92.50)	3 (7.50)
c^2^ value		1.053
*P* value		0.305

Three months after surgery, the scores of the Hunt-Hess and modified Rankin scale in the two groups were significantly lower than those before surgery, and the degree of decrease in the observation group was significantly lower than that in the control group, with a statistically significant difference (*p*<0.05) Table-III

**Table-III:** Comparison of the scores of Hunt-Hess and modified Rankin scale between the two groups (*χ̅*±*S*).

Group	Hunt-Hess		Rankin score	

	Before surgery	Three months after surgery	Before surgery	Three months after surgery
Control group (n=40)	25.15±1.25	13.05±1.34	4.15±0.74	2.30±0.56
Observation group (n=40)	25.08±1.27	8.05±1.24	3.95±0.68	1.80±0.69
*t* value	0.266	17.334	1.265	3.558
*P* value	0.791	0.000	0.210	0.001

The good prognosis rate of the observation group was higher than that of the control group, with a statistically significant difference between the two groups (*p<*0.05) Table-IV The incidence of complications in the observation group was 12.50%, which was significantly lower than 32.50% in the control group (*p<*0.05) Table-V.

**Table-IV:** Comparison of good prognosis rate between the two groups [n (%)].

Group	GOS score					Good rate

	1 point	2 points	3 points	4 points	5 points	
Control group (n=40)	0 (0.00)	3 (7.50)	11 (27.50)	18 (45.00)	8 (20.00)	26 (65.00)
Observation group (n=40)	0 (0.00)	1 (2.50)	5 (12.50)	20 (50.00)	14 (35.00)	34 (85.00)
χ² value						4.267
*P* value						0.039

**Table-V:** Comparison of surgical complications between the two groups [*n*, (%)].

Group	n	Cerebral vasospasm	Rebleeding	Cerebral infarction	Neurological dysfunction	Intracranial infection	Total
Control group	40	4 (10.00)	2 (5.00)	2 (5.00)	3(7.50)	2(5.00)	13 (32.50)
Observation group	40	2 (5.00)	1(2.50)	1(2.50)	1(2.50)	0 (0.00)	5 (12.50)
c^2^ value							4.588
P value							0.032

## DISCUSSION

In this study, the clinical effects of craniotomy and interventional embolization in the treatment of anterior circulation aneurysms were compared. The blood loss and hospitalization time of patients in the observation group were significantly lower than those in the control group. Interventional treatment of anterior circulation aneurysms is performed by embolization through blood vessels to the focus under fluoroscopy, which effectively avoids brain tissue damage caused by craniotomy and surgery, indicating that interventional embolization has less trauma, less blood loss and faster postoperative recovery. Three months after surgery, Hunt-Hess score and modified Rankin score decreased significantly in the observation group compared with the control group, suggesting that the improvement level of neurological function and quality of life of patients after interventional embolization was better than that of craniotomy embolization, which was the same as that of Liu et al.^8^

With the acceleration of the aging society in China, there is an obvious increase in the number of patients with anterior circulation aneurysms.^9^,^10^ Most scholars harbor the idea that the pathogenesis of anterior circulation aneurysms includes two factors: congenital factors such as hemodynamic changes and vascular wall lesions, and acquired factors such as atherosclerosis, vasculitis and severe hypertension.^11^,^12^ Once the aneurysm ruptures, it will cause subarachnoid hemorrhage and increase intracranial pressure. If not treated in time, shock and encephalopathy may occur, resulting in a sharp deterioration of the patient’s condition and a high disability and mortality rate.^13^-^16^ Therefore, it is of great significance to treat anterior circulation aneurysms early.^17^

Currently, craniotomy clipping and endovascular interventional embolization are the preferred options for the treatment of anterior circulation aneurysms. Specifically, craniotomy clipping can clip aneurysms under direct vision, which has a definite curative effect and a high success rate. However, this operation needs to be performed after the relatively stable condition of patients, with large surgical trauma, easy damage to the brain tissue around the tumor during the operation, and sequelae, which brings great pain to patients.^18^ In contrast, interventional embolization, as a minimally invasive operation, boasts the advantages of a small wound, light injury, quick recovery and good prognosis. It not only guarantees its safety but also has a high success rate. However, interventional embolization has its drawbacks such as being demanding, risky, expensive and poor operation timing.

In recent years, more and more attention has been paid to the application of endovascular interventional embolization in the treatment of anterior circulation aneurysms, and it has become one of the important surgical methods for minimally invasive treatment of anterior circulation aneurysms.^19^,^20^ In this study, the good rate of GOS score in the observation group was significantly higher than the control group. The incidence of postoperative complications in the observation group was lower than that in the observation group. All these suggest that interventional embolization in the treatment of anterior circulation aneurysms can significantly improve the prognosis of patients and effectively reduce complications. By analyzing the reasons, we concluded that interventional embolization can effectively avoid brain tissue damage and reduce the risk of complications due to operation without direct contact with brain tissue. Moreover, interventional embolization is a minimally invasive operation that uses a special catheter system to place the coil into the artery cavity of the patient and fill it up, which improves the surgical effect. Supplemented with a short postoperative bedtime to promote early recovery of patients.

### Limitations:

It includes a single-center study with small sample size. There may be some selection bias and short follow-up time. To address this, further research is still needed on the long-term efficacy of interventional embolization and the long-term prognosis of patients.

## CONCLUSION

Interventional embolization can significantly ameliorate the neurological function and quality of life of patients with the advantages of short operation time and short hospitalization time, and improve the prognosis of patients, and a low incidence of complications. It is worthy of vigorous promotion and application in clinical practice.

### Authors’ Contributions:

**BL and GY** carried out the studies, participated in collecting data, drafted the manuscript’ are responsible and accountable for the accuracy and integrity of the work.

**XW and** **HW** performed the statistical analysis and participated in its design.

**NZ** participated in acquisition, analysis, or interpretation of data and draft the manuscript.

All authors read and approved the final manuscript.
